# Centenarians’ offspring as a model of healthy aging: a reappraisal of the data on Italian subjects and a comprehensive overview

**DOI:** 10.18632/aging.100912

**Published:** 2016-03-12

**Authors:** Laura Bucci, Rita Ostan, Elisa Cevenini, Elisa Pini, Maria Scurti, Giovanni Vitale, Daniela Mari, Calogero Caruso, Paolo Sansoni, Flaminia Fanelli, Renato Pasquali, Paola Gueresi, Claudio Franceschi, Daniela Monti

**Affiliations:** ^1^ Department of Experimental, Diagnostic and Specialty Medicine (DIMES) and Interdepartmental Centre “L. Galvani” (CIG), University of Bologna, 40126 Bologna, Italy; ^2^ Department of Clinical Sciences and Community Health (DISCCO), University of Milan, 20122 Milan, Italy; ^3^ Laboratory of Endocrine and Metabolic Research, IRCCS Istituto Auxologico Italiano, Cusano Milanino (MI) 20095, Italy; ^4^ Geriatric Unit, Fondazione Ca' Granda, IRCCS Ospedale Maggiore Policlinico, 20122 Milan, Italy; ^5^ Department of Pathobiology and Medical and Forensic Biotechnologies, University of Palermo, 90134 Palermo, Italy; ^6^ Department of Clinical and Experimental Medicine, University of Parma, 43126 Parma, Italy; ^7^ Endocrinology Unit, and Department of Medical and Surgical Sciences and Center for Applied Biomedical Sciences, St Orsola-Malpighi Hospital, University of Bologna, 40138 Bologna, Italy; ^8^ Department of Statistical Sciences “Paolo Fortunati”, University of Bologna, 40126 Bologna, Italy; ^9^ Department of Clinical, Experimental and Biomedical Sciences, University of Florence, 50134 Florence, Italy

**Keywords:** aging/longevity, centenarians’ offspring, health status, inflammaging, age-related diseases

## Abstract

Within the scenario of an increasing life expectancy worldwide it is mandatory to identify determinants of healthy aging. Centenarian offspring (CO) is one of the most informative model to identify trajectories of healthy aging and their determinants (genetic and environmental), being representative of elderly in their 70^th^ whose lifestyle can be still modified to attain a better health. This study is the first comprehensive investigation of the health status of 267 CO (mean age: 70.2 years) and adopts the innovative approach of comparing CO with 107 age-matched offspring of non-long-lived parents (hereafter indicated as NCO controls), recruited according to strict inclusion demographic criteria of Italian population. We adopted a multidimensional approach which integrates functional and cognitive assessment together with epidemiological and clinical data, including pro- and anti-inflammatory cytokines and adipokines, lipid profile, and insulin resistance. CO have a lower prevalence of stroke, cerebral thrombosis-hemorrhage, hypertension, hypercholesterolemia, and other minor diseases, lower BMI and waist circumference, a better functional and cognitive status and lower plasma level of FT4 compared to NCO controls. We conclude that a multidimensional approach is a reliable strategy to identify the health status of elderly at an age when interventions to modify their health trajectory are feasible.

## INTRODUCTION

Centenarians, i.e subjects who reached extreme longevity, are an extraordinary model to study human longevity and healthy aging [1-4]. However, the study of centenarians have some limitations (rarity, lack of an age-matched control group and frailty related to extreme age). Some of these difficulties can be overcome by studying centenarians’ offspring (CO) who are one generation (about 20-30 years) younger than centenarians and are representative of elderly whose lifestyle can be still modified to attain a better health. Moreover, CO allow to study both genetic and environmental/lifestyle determinants of healthy aging. In fact, human longevity seems to cluster in families enriched in long-lived parents and ancestors, and parents/mothers who later will become centenarians likely adopt more healthy lifestyle for their children [5]. An impressive and coherent series of epidemiological data from different populations (White Americans from New England, Mormons from Utah, Ashkenazi Jewish living in USA, Icelanders, Japanese from Okinawa, and Netherlanders from Leiden) points out the presence of a strong familiar and genetic component of human longevity. Overall, these studies suggest that relatives (parents, siblings and offspring) of long-lived subjects have a significant survival advantage, a higher probability to have been or to become long-lived and a lower risk to undergo to major age-related diseases, such as cardio- and cerebral-vascular diseases (CVD), diabetes, and cancer [6-17], associated with a favourable lipoprotein profile (larger HDL and LDL particle size) and increased homozygosity for the 405 valine allele (V allele) in the *CETP* gene (Cholesteryl Ester Transfer Protein) [18], and the −641 C allele in *APOC3* gene [19]. Therefore, relatives of centenarians and in particular CO represent an informative model to study biological and genetic factors involved in human healthy aging and longevity. Despite the interest of the CO model to study determinants and trajectories of healthy aging, a comprehensive and deep characterization of the CO phenotype is still lacking. Accordingly, we adopted a multidimensional approach which integrates functional and cognitive assessment together with epidemiological and clinical data, including pro- and anti-inflammatory cytokines and adipokines, lipid profile, and insulin resistance. Moreover, we paid particular attention to the control group to be compared with CO. Indeed, at variance with previous studies (see Supplemental Table 1) we made an effort to recruit unrelated age-matched elderly born from non-long lived parents. Indeed, CO were compared to age-matched elderly whose parents (both) were born in the same birth cohort of centenarians but who died before reaching the age threshold over which subjects can be classified as long-lived, according to strict Italian demographic data [20]. These subjects are hereafter indicated as “NCO controls”. Within this scenario, the present study aimed at investigating the health status of a consistent group of Italian CO using for the first time a multidimensional approach (epidemiological and clinical data; functional and cognitive measures such as ADL, SMMSE, chair stand and handgrip strength tests; pro- and anti-inflammatory cytokines and adipokines, and major biochemical risk factors for age-related diseases) [4,21-28], in comparison to a group of unrelated NCO controls concomitantly assessed by the same protocol.

## RESULTS

Table 1 compares the general and anthropometric characteristics of CO and NCO controls in the whole population and in males and females, separately. CO showed higher education level (p=.048 for entire population; p=.009 for females) and a lower proportion of married individuals, particularly among females (p=.001 for entire population; p=.046 for females). Life-style characteristics were not different between the two groups. Intriguingly, significant differences between CO and NCO controls emerged from the evaluation of anthropometric measurements. In the entire population, CO had lower weight (p<.001), smaller waist (p=.003) and hip circumferences (p=.061), lower waist/hip ratio (p=.014) and lower BMI (p=.002) underlining that they were leaner than NCO controls; in fact a lower proportion of overweight and obese individuals was present within the group of CO (p=.010). The waist/height ratio was also significantly lower in CO compared to NCO controls (p=.017) despite the two groups exhibited the same height. These anthropometric traits show a gender distinction. Female CO showed a significantly lower weight (p=.001), waist (p=.005) and hip (p=.013) circumferences, waist/height ratio (p=.016), BMI (p=.006) and a lower proportion of obese and overweight subjects (p=.008) in comparison to female NCO controls. In contrast, no differences were evidenced in males.

**Table 1:** Demographic, lifestyle and anthropometric features of centenarians’ offspring (CO) and offspring of non-long-lived parents (NCO)

	Total, N=374			Men, N=163			Women, N=211		
	CO **N=267**	NCO **N=107**	p-value	CO **N=108**	NCO **N=551**	p-value	CO **N=159**	NCO **N=52**	p-value
Age, mean (SD)	70.2 (6.6)	71.1 (6.0)	0.230	70.2 (6.3)	70.4 (6.3)	0.865	70.2 (6.8)	71.7 (5.7)	0.107
Education, years, mean (SD)	11.2 (5.0)	10.1 (4.3)	**0.048**	11.3 (5.2)	10.8 (4.8)	0.608	11.1 (4.9)	9.4 (3.7)	**0.009**
**Marital Status**									
Never married, n (%)	43 (16.1)	3 (2.8)	**0.001**	12 (11.1)	0 (0.0)	0.058	31 (19.5)	3 (5.8)	**0.046**
Married, n (%)	159 (59.6)	82 (76.6)		84 (77.8)	48 (87.3)		75 (47.2)	34 (65.4)	
Divorced, n (%)	14 (5.2)	3 (2.8)		6 (5.6)	2 (3.6)		8 (5.0)	1 (1.9)	
Widow/widower, n (%)	51 (19.1)	19 (17.8)		6 (5.6)	5 (9.1)		45 (28.3)	14 (26.9)	
**Life-style**									
Current smokers, n (%)	38 (14.3)	18 (16.8)	0.544	13 (12.1)	8 (14.5)	0.667	25 (15.8)	10 (19.2)	0.567
Former smokers, n (%)	102 (45.1)	40 (45.5)	0.959	55 (57.9)	28 (60.9)	0.736	47 (35.9)	12 (28.6)	0.385
Daily alcohol consumption, n (%)	137 (52.5)	57 (54.3)	0.756	72 (67.9)	35 (64.8)	0.693	65 (41.9)	22 (43.1)	0.880
**Anthropometric features**									
Weight, Kg, mean (SD)	69.7 (13.2)	75.3 (12.8)	**<0.001**	77.0 (12.3)	79.5 (12.4)	0.223	64.6 (11.4)	70.8 (11.8)	**0.001**
Height, cm, mean (SD)	161.8 (8.9)	163.6 (8.8)	0.090	168.7 (7.0)	169.0 (6.7)	0.781	157.1 (6.8)	157.7 (6.8)	0.594
Waist circumference, cm mean (SD)	90.9 (13.0)	95.3 (11.4)	**0.003**	97.2 (10.8)	98.0 (10.1)	0.651	86.6 (12.6)	92.3 (12.0)	**0.005**
Hip circumference, cm mean (SD)	103.4 (10.1)	105.5 (8.8)	0.061	103.5 (8.8)	103.5 (8.0)	0.969	103.4 (10.8)	107.6 (9.2)	**0.013**
Waist/hip, mean (SD)	0.88 (0.1)	0.90 (0.1)	**0.014**	0.94 (0.06)	0.9 (0.06)	0.610	0.84 (0.07)	0.86 (0.07)	0.070
Waist/height, mean (SD)	0.56 (0.08)	0.58 (0.07)	**0.017**	0.58 (0.06)	0.58 (0.06)	0.691	0.55 (0.09)	0.59 (0.08)	**0.016**
BMI, Kg/m^2^, mean (SD)	26.5 (4.4)	28.1 (4.2)	**0.002**	26.9 (3.5)	27.8 (3.9)	0.125	26.3 (5.0)	28.5 (4.5)	**0.006**
Normal (BMI<25), n (%)	103 (39.5)	24 (22.9)	**0.010**	34 (32.1)	14 (25.5)	0.647	69 (44.5)	10 (20.0)	**0.008**
Overweight (25≤BMI<30), n (%)	109 (41.8)	54 (51.4)		53 (50.0)	29 (52.7)		56 (36.1)	25 (50.0)	
Obese (BMI≥30), n (%)	49 (18.8)	27 (25.7)		19 (17.9)	12 (21.8)		30 (19.4)	15 (30.0)	

Abbreviations: SD, standard deviation; %, percentage.

*Former smokers were calculated on the number of subjects who had declared of not smoking at the moment of the interview.

Table 2 shows the functional and cognitive status of CO and NCO controls. Considering the autonomy and self-sufficiency a significantly higher proportion of CO was completely continent (p=.018), able to walk 500 meters (p=.034) and to climb up and down the stairs without anyone's help (p=.002) compared to NCO controls. This leads to a lower use of walking aids (such as a wheelchair, cane, etc.) in the group of CO (p=.017). The distribution of subjects across handgrip strength quartiles showed that a higher prevalence of CO was in the first “best performing” quartile (p=.038) and a higher proportion of them was able to perform chair stand test (p<.001) while, among those who were able, the time spent to perform the exercise was not different between CO and NCO controls (data not shown). Once again, female CO showed a significantly better functional status compared to female NCO controls (Table 2). On the whole, although both groups are free from clinically overt disability, CO were more functionally fit than controls.

**Table 2:** Functional and cognitive status of centenarians’ offspring (CO) and offspring of non-long-lived parents (NCO)

	Total, N=374			Men, N=163			Women, N=211		
	CO **N=267**	NCO **N=107**	p-value	CO **N=108**	NCO **N=55**	p-value	CO **N=159**	NCO **N=52**	p-value
**Autonomy and self-sufficiency**									
ADL 5 items, mean (SD)	5 (0.1)	4.9 (0.6)	0.127	5 (0.0)	5.0 (0.3)	0.322	5.0 (0.0)	4.8 (0.8)	0.197
Completely continent, n (%)	240 (90.2)	87 (81.3)	**0.018**	99 (92.5)	50 (90.9)	0.720	141 (88.7)	37 (71.2)	**0.003**
IADL, mean (SD)	7.9 (0.5)	7.7 (1.1)	**0.040**	7.9 (0.4)	7.7 (0.9)	0.169	7.9 (0.6)	7.7 (1.3)	0.163
Ability to walk 500 meters, n (%)	254 (95.5)	95 (89.6)	**0.034**	107 (99.1)	53 (96.4)	0.223	147 (93.0)	42 (82.4)	**0.024**
Ability to go up and down the stairs, n (%)	228 (85.4)	76 (71.7)	**0.002**	101 (93.5)	47 (85.5)	0.144	127 (79.9)	29 (56.9)	**0.001**
Use of aids, n (%)	9 (3.4)	10 (9.4)	**0.017**	2 (1.9)	4 (7.3)	0.181	7 (4.4)	6 (11.8)	0.058
**Handgrip strength test**									
1^st^ quartile, n (%)	70 (26.8)	18 (17.1)	**0.038**	25 (23.1)	15 (27.8)	0.103	45 (29.4)	3 (5.9)	**0.007**
2^nd^ quartile, n (%)	64 (24.5)	19 (18.1)		28 (25.9)	5 (9.3)		36 (23.5)	14 (27.5)	
3^rd^ quartile, n (%)	69 (26.4)	37 (35.2)		31 (28.7)	19 (35.2)		38 (24.8)	18 (35.3)	
4^th^ quartile, n (%)	58 (22.2)	31 (29.5)		24 (22.2)	15 (27.8)		34 (22.2)	16 (31.4)	
Ability to perform chair stand test, n (%)	251 (96.2)	87 (84.5)	**<0.001**	103 (97.2)	46 (88.5)	**0.027**	148 (95.5)	41 (80.4)	**0.002**
**Cognitive function**									
SMMSE, mean (SD)	29.0 (1.5)	28.6 (2.5)	**0.043**	29.1 (1.2)	28.9 (1.3)	0.360	29.0 (1.7)	28.3 (3.3)	0.145
**Mood**									
GDS, mean (SD)	3.0 (3.0)	2.7 (2.6)	0.206	2.7 (2.7)	2.0 (2.3)	0.156	3.3 (3.2)	3.4 (2.7)	0.888

The scores obtained in the ADL and GDS tests were similar between two groups (Table 2) while CO attained a better mean score in IADL (p=.040) and SMMSE (p=.043).

Table 3 shows the relative prevalence of some age-related diseases in CO and NCO after adjusting for possible confounders such as age, gender, education, marital status and BMI. CO have had a reduced pre-valence of stroke/cerebral thrombosis-hemorrhage (− 80%; p=.022) and a trend towards a lower prevalence of cancer (malign: - 47%; p=.071; benign: - 58%; p=.072). At the moment of the interview, CO had a significantly lower prevalence of hypertension (− 52%; p=.006), hypercholesterolemia (− 44%; p=.025) and a marginally significant lower prevalence of COPD (− 61%; p=.073) and osteoporosis (− 48%; p=.051). CO reported a lower prevalence of a series of ailments, such as glaucoma, gastritis, prostatic hypertrophy, etc, collectively indicated as “other diseases” (− 52%; p=.004).

**Table 3:** Functional and cognitive status of centenarians’ offspring (CO) and offspring of non-long-lived parents (NCO)

	N	CO	N	NCO	OR (95% CI)	p-value
**Past diseases**						
Pneumonia, n (%)	267	65 (24.3)	106	22 (20.8)	1.277 (0.723 - 2.254)	0.400
Myocardial Infarction, n (%)	267	12 (4.5)	106	12 (11.3)	0.489 (0.201 - 1.194)	0.116
Stroke, cerebral thrombosis-haemorrhage, n (%)	266	4 (1.5)	105	8 (7.6)	0.200 (0.051 - 0.789)	**0.022**
Malign tumor, n (%)	267	26 (9.7)	106	18 (17.0)	0.533 (0.269 - 1.056)	0.071
Benign tumor, n (%)	267	11 (4.1)	106	10 (9.4)	0.419 (0.162 - 1.083)	0.072
Hip fracture, n (%)	267	5 (1.9)	106	4 (3.8)	0.535 (0.136 - 2.113)	0.372
Spinal collapse, n (%)	267	8 (3.0)	106	6 (5.7)	0.404 (0.122 - 1.336)	0.137
**Current diseases**						
Heart failure, n (%)	267	2 (0.7)	106	4 (3.8)	0.276 (0.047 - 1.611)	0.153
Irregular heart rhythm, n (%)	267	29 (10.9)	107	20 (18.7)	0.584 (0.301 - 1.131)	0.110
Hypertension, n (%)	267	109 (40.8)	107	68 (63.6)	0.485 (0.288 - 0.816)	**0.006**
COPD, n (%)	267	9 (3.4)	106	9 (8.5)	0.394 (0.143 - 1.089)	0.073
Dementia, n (%)	267	0 (0.0)	106	1 (0.9)	na	na
Depression/Anxiety, n (%)	267	41 (15.4)	106	22 (20.8)	0.669 (0.362 - 1.239)	0.201
Osteoporosis, n (%)	267	44 (16.5)	106	24 (22.6)	0.520 (0.270 - 1.002)	0.051
Diabetes, n (%)	267	26 (9.7)	106	12 (11.3)	1.042 (0.462 - 2.352)	0.921
Hypothyroidism, n (%)	267	25 (9.4)	106	8 (7.5)	0.988 (0.400 - 2.437)	0.979
Hyperthyroidism, n (%)	267	3 (1.1)	106	4 (3.8)	0.290 (0.053 - 1.593)	0.154
Hypercholesterolemia, n (%)	267	91 (34.1)	106	46 (43.4)	0.563 (0.342 - 0.929)	**0.025**
Chronic renal insufficiency, n (%)	267	3 (1.1)	106	5 (4.7)	0.273 (0.052 - 1.423)	0.123
**^a^**Other diseases, n (%)	265	85 (32.1)	107	53 (49.5)	0.484 (0.296 - 0.793)	**0.004**
**Drugs use, n (%)**						
0 drugs, n (%)	267	58 (21.7)	106	7 (6.6)	1	
1-3 drugs, n (%)	267	148 (55.4)	106	48 (45.3)	0.375 (0.156 - 0.897)	**0.028**
4 or more drugs, n (%)	267	61 (22.8)	106	51 (48.1)	0.136 (0.053 - 0.347)	**<0.001**
Cardiovascular Therapy, n (%)	267	54 (20.2)	106	36 (34.0)	0.493 (0.299 - 0.813)	**0.005**
Hypotensive Therapy, n (%)	267	106 (39.7)	106	68 (64.2)	0.368 (0.231 - 0.587)	**<0.001**
Antidiabetic Therapy, n (%)	267	15 (5.6)	106	10 (9.4)	0.571 (0.248 - 1.316)	0.188
Lipid-lowering Therapy, n (%)	267	47 (17.6)	106	40 (37.7)	0.353 (0.213 - 0.583)	**<0.001**

Prevalence of past and current diseases was adjusted for age, gender, education, marital status and BMI.

a The following conditions are included: prostatic hypertrophy, cataract and glaucoma, gastritis, hiatus hernia, cholelithiasis, allergies, hemicranias, haemorrhoids, etc. na: not assessed.

With regard to the current pharmacological therapy, a lower percentage of CO underwent a multi-drug therapy with more than 4 drugs (p<.001) when compared to NCO controls. In particular, CO were less treated with cardiovascular (p=.005), hypotensive (p<.001) and lipid-lowering (p<.001) drugs (Table 3).

Table 4 shows conventional CVD risk factors, metabolic and inflammatory mediators in CO and NCO controls. CO had higher levels of total (p=.037) and LDL (p=.003) cholesterol, and lower levels of HDL cholesterol (p=.019) with a consequent worse total/HDL cholesterol ratio (p=.001) showing an apparently unfavourable lipid profile in comparison to NCO controls. Other risk factors, such as triglycerides and inflammatory markers like CRP, A-SAA, and IL-6, as well as levels of adiponectin, leptin, resistin and TNF-α did not change between CO and NCO controls (Table 4). Finally, glycaemia, insulin and insulin resistance index (HOMA-IR index) were similar between the two groups. Analyzing thyroid hormones, we observed that CO had a significantly lower levels of free thyroxin (FT4, p=.033) in respect to NCO controls.

**Table 4:** Functional and cognitive status of centenarians’ offspring (CO) and offspring of non-long-lived parents (NCO)

	CO **N=266**	NCO **N=105**	p-value
**Conventional Risk Factors**			
Total cholesterol, mg/dl	203.0 (177.0 – 231.0)	195.0 (171.5 – 217.0)	**0.037**
LDL cholesterol, mg/dl	119.3 (98.2 – 150.0)	112.0 (88.0 – 135.6)	**0.003**
HDL cholesterol, mg/dl	51.0 (42.0 – 63.0)	56.0 (45.5 – 74.0)	**0.019**
Total/HDL cholesterol ratio	4.0 (3.1 – 4.8)	3.4 (2.7 – 4.2)	**0.001**
Triglycerides, mg/dl	111.5 (86.0 – 156.0)	104.5 (79.0 – 146.0)	0.175
Albumin, mg/dl	4.2 (3.4 – 4.5)	4.1 (3.4 – 4.4)	0.445
CRP, mg/L	1.3 (0.8 – 3.0)	1.6 (0.8 – 3.2)	0.341
A-SAA protein, mg/ml	92.0 (44.9 – 197.3)	85.3 (50.7 – 177.7)	0.959
IL-6, pg/ml	16.7 (10.0 – 34.0)	19.1 (10.9 – 31.4)	0.500
**Metabolic mediators**			
Adiponectin, μg/ml	34.0 (19.3 – 56.3)	30.6 (18.3 – 56.4)	0.496
Leptin, ng/ml	14.3 (7.3 – 31.8)	14.3 (6.7 – 32.4)	0.991
Resistin, ng/ml	9.2 (7.0 – 12.2)	9.1 (7.0 – 12.4)	0.964
TNF-α, pg/ml	10.3 (2.8 – 31.9)	11.0 (3.8 – 31.5)	0.655
**Insulin resistance markers**			
Glycaemia, mmol/L	4.8 (4.4 – 5.4)	4.9 (4.6 – 5.5)	0.095
Insulin, μIUg/ml	10.4 (6.2 – 14.9)	10.5 (6.9 – 14.1)	0.704
HOMA-IR index	2.2 (1.3 – 3.5)	2.5 (1.5 – 3.6)	0.393
^a^**Thyroid Hormones**			
Free triiodothyronine (FT3), pg/ml	3.0 (2.8 – 3.3)	3.0 (2.8 – 3.3)	0.922
Free thyroxin (FT4), ng/ml	11.5 (10.4 – 12.6)	11.9 (10.9 – 12.8)	**0.033**
TSH, mIU/ml	1.8 (1.3 – 2.5)	1.8 (1.3 – 2.4)	0.798
T3/T4 ratio	0.3 (0.2 – 0.3)	0.3 (0.2 – 0.3)	0.101

a Subjects under thyroid therapy were excluded from the analysis. Data are expressed as median (min-max) between parenthesis.

p-value was derived from Mann-Whitney test.

## DISCUSSION

Human longevity clusters in families enriched in long-lived parents and ancestors. Several studies were concordant in indicating that CO have a longer survival and are significantly healthier compared with age-matched controls, thus representing a powerful and informative model to study the determinants of healthy aging and longevity [7-9]. Our data show that Italian CO have a better functional and cognitive fitness (higher IADL and SMMSE scores; ability of walking 500 meter; going up and down the stairs; performing Chair Stand test; displaying higher grip strength) and were leaner than controls in terms of weight, hip circumference, BMI, overweight and obesity prevalence, confirming previous findings in CO living in non-European countries (e.g. USA) [7] and belonging to a different ethnicity (Ashkenazi Jewish) [10]. Our new data on central adiposity (waist circumference, waist/hip ratio and waist/height ratio) give further support to the notion that CO do have a peculiar body anthropometrical characteristics which emerged in all the different cohorts so far examined. Central adiposity is a strong predictors of cardiovascular diseases (CVD) and mortality risk [29-33]. Our data and those of previous studies suggest that being leaner and having less visceral adiposity at age 70 is likely a strong contributor to the better health status of CO, in comparison to NCO. The animal model counterpart of this observation in humans is represented by data showing that *ad libitum*-fed rats with selective removal of visceral fat had a significant increase in mean and maximum lifespan and a significant reduction in the incidence of severe renal diseases compared to *ad libitum*-fed rats without visceral fat removal [34]. On the whole, the data in humans and animal model are concordant in supporting the notion that fat mass reduction, mainly visceral fat, may be one of the possible anti-aging mechanisms able to modulate mammalian longevity [34]. On the contrary, a great number of cross-sectional and longitudinal studies indicate that weight gain, increased BMI and obesity contribute to a decline in physical and cognitive activity [35-37], and that a poor performance in Chair Stand tests and the loss of muscle strength are associated with adverse health outcomes in older persons [38-40].

An increase in body fat, particularly visceral fat and high BMI have been associated with insulin resistance [41-42], higher risk for diabetes, cardiovascular disease [43], hypertension and metabolic disorders [44]. Consistently, we found that Italian CO not only showed a better physical status, but had a lower prevalence of pathological conditions (myocardial infarction, stroke, cerebral thrombosis/hemorrhage, arrhythmia and hypertension) than NCO controls, as previously reported in New England and Ashkenazi population [7,10]. These findings are supported by data regarding pharmacological therapy. Italian CO used fewer drugs and a lower percentage of them was under cardiovascular, hypotensive and lipid-lowering therapy compared to NCO controls. The reduced number of CO under pharmacological therapy could explain their apparent unfavorable lipid profile (higher levels of total and LDL cholesterol, total/HDL cholesterol ratio and lower levels of HDL cholesterol) in comparison to NCO controls. However, it is worth noting that the values reported for CO are within the normal range and are not indicative of CVD risk. At variance with a previous study [8,10], the prevalence of diabetes in Italian CO was similar to that of NCO controls. We surmise that differences regarding environmental and anthropological context, cultural habits and lifestyle factors (e.g. diet) between Italian CO and those living in other continents (USA) and belonging to different ethnicities (Ashkenazi Jewish) may be largely responsible for this apparent discrepancy.

One of the major features of the aging process is the presence of a chronic low-grade inflammatory status (inflammaging) which contributes to the development of different pathological conditions [28,45-49]. This peculiar inflammatory activity, leading to long-term tissue damage has been found to be related to mortality risk for all causes in older persons [50]. In this study, parameters related to inflammaging were similar between CO and NCO controls, suggesting that significant differences might appear later in life, and longitudinal studies are needed to clarify this point.

CO obtained higher scores in the handgrip test compared to controls. In a previous work conducted on the same subjects, we observed lower levels of IGF-1 in CO in comparison to NCO [51]. Data from the present paper highlighted that, although having lower levels of IGF-1, CO did not show the typical negative aspects related to a low IGF-1 levels in elderly such as loss of muscle mass and power [52] and higher risk of CVD [53-54]. Therefore, the role of this potent anabolic hormone in the genesis of the aging phenotype remains controversial and its complex function during specific stages of the life span should be deeply explored [51,55].

Several changes in thyroid function are frequently observed in the elderly and some studies have shown that centenarians and their relatives have reduced levels of FT3, FT4 and TSH [56,57]. Findings of this study did not show any significant difference between CO and NCO in the levels of FT3, TSH thyroid hormones and in T3/T4 ratio. The levels of FT4 appear significantly lower in CO, but the difference is not so relevant to have a biological meaning. The subtle thyroid hypofunction reported in literature may onset in more advanced decades of life in CO as an adaptive mechanism to reset the hormonal milieu favouring successful ageing and longevity.

This study has several strengths. First of all, the peculiar experimental model including centenarians’ offspring and age-matched offspring of non-long-lived parents born in the same cohort of centenarians. It is worth noting that, in comparison with other papers, in this study, controls are selected with a more accurate method envisaging strict demographic criteria. However, even if the identification and the recruitment of offspring of both non-long-lived parents was very demanding, the size of the cohort studied is fully comparable to previous researches (see Supplemental Table 1). Secondly, all participants have been well characterized: medical history was accurately documented and several anthropometric, inflammatory and metabolic parameters were assessed concomitantly to provide a comprehensive picture of their health status, from a molecular level (for example through the evaluation of inflammatory and metabolic mediators) to a systemic/organism level (for example through the evaluation of the ability to perform physical tests). This is worthwhile, since the aging process differently affects each organ of the body and also every tissue and cell type composing the organ, giving rise to the so-called “aging mosaic” [58]. Therefore, in order to disentangle the complexity of the aging process it is necessary to study the organism by means of a wide approach.

## FUTURE PERSPECTIVES

Since centenarians’ offspring emerged as an excellent model to study human aging and longevity, longitudinal studies are mandatory to better understand their diseases’ susceptibility and outcomes. Moreover, even if centenarians’ offspring and controls have been recruited in the same geographic area and we can suppose they followed a similar diet, the evaluation of dietary habits will be essential in future studies. In fact, dietary behavior and nutrients intake have been associated with occurrence, development and outcomes of major age-related diseases having a role in determining the overall health status of elderly people impinging upon inflammation, oxidative stress and gut microbiota composition.

## METHODS

### Study design and participants

A total of 374 subjects were enrolled in five Italian cities (Bologna, Milan, Florence, Parma and Palermo) and surrounding areas. The group of CO consisted of 267 subjects [n=108 males, mean age (SD) = 70.2 (6.3) years and n=159 females, mean age (SD) = 70.2 (6.8) years], having a centenarian parent born between 1900-1908. The group of NCO controls consisted of 107 age-matched offspring [n=55 males, mean age (SD) = 70.4 (6.3) years and n=52 females, mean age (SD) = 71.7 (5.7) years], having both parents born in the same birth cohort of centenarians (1900-1908), but dead before the threshold age over which subjects were classified “long-lived” as previously described [20].

The lists of centenarians and their offspring (CO) were obtained by the Register Office, while the offspring of non-long-lived parents (NCO) were identified by checking the birth and death dates of their parents in paper population records from Registers Office. All participants signed the informed consent before undergoing the questionnaires, measurements and blood sampling. The study protocol was approved by the Ethical Committee of Sant'Orsola-Malpighi University Hospital (Bologna, Italy).

### Measurement tools

Anthropometric measures were performed while participants wore light dresses and no shoes. Body weight was measured using the balance SECA Mod. 761 calibrated in kilograms. Height was measured with the subject standing barefoot with feet together, using an anthropometer calibrated in centimeters. Waist circumference was measured using a flexible steel tape at the end of exhalation, by wrapping the tape at the level of the iliac crest and umbilicus, with the subject standing [59]. Hip circumference was measured at the level of maximal protrusion of the gluteal muscles. Waist-hip ratio was calculated as the ratio of waist circumference to hip circumference. Waist-height ratio was calculated as the ratio of waist circumference to standard height. Body mass index (BMI) was calculated as weight in kilograms divided by the square of height in meters (kg/m^2^).

Functional status was assessed by ADL-Activities of Daily Living scale (scores ranging from 0 [all functions lost] to 5 [all functions preserved]) [60]. Continence was analyzed separately [61]. Ability in home management was assessed by IADL-Instrumental Activities of Daily Living scale (scores ranging from 0 [all functions lost] to 8 [all functions preserved]) [62]. Physical performance was assessed by self-reported data regarding the ability to walk 500 meters, to go up and down the stairs and the use of aids. Handgrip Strength Test and Chair Stand Test were performed, the former to measure the maximum isometric strength of the hand and forearm muscles and the latter to evaluate leg strength and endurance measuring the time it takes to perform 5 repetitions of sit-to-stand [63]. Handgrip strength was measured using a hand-held dynamometer (SMEDLYS’ dynamometer, Scandidact, Kvistgaard, Denmark) for two performances with each hand. The best performance was selected for the analysis and scores were divided into quartiles distinguishing between men and women. The first quartile included the “best-performing” participants. Regarding the Chair Stand Test, participants were divided into two groups (able or unable to complete the test). Cognitive status was assessed by SMMSE-Standardized Mini-Mental State Examination test [64] and scores were adjusted by age and education according to Magni [65]. Mood was investigated by GDS-Geriatric Depression Scale short form (15 items) [66]. History of past and current diseases was accurately collected by checking the participants' medical documentation and addressing the major age-related pathologies: pneumonia, myocardial infarction, stroke/cerebral thrombosis-hemorrhage, cancer, benign tumor, hip fracture, spinal collapse, heart failure, cardiac arrhythmia, high blood pressure, chronic obstructive pulmonary disease (COPD), dementia, depression/anxiety, osteoporosis, diabetes mellitus, hypo and hyperthyroidism, hypercholesterolemia and chronic renal insufficiency.

Current use of medication (including inspection of the drugs by the interviewer) was recorded and drugs grouped in 4 main therapies: cardiovascular (anti-arrhythmic and/or vasodilatator and/or thrombolytic drugs), hypotensive (calcium channel blockers and/or ACE inhibitors and/or diuretics and/or beta blockers), antidiabetic (sulfonylureas and/or biguanides and/or insulin and insulin analogues and/or other oral antidiabetic association), and lipid-lowering (statins and/or fibrates and/or other hypolipidemic agents) therapy.

### Laboratory measurements

Overnight fasting blood samples were obtained in the morning. Serum was obtained after clotting and centrifugation at 760 g for 20 min at 4°C, rapidly frozen and stored at −80°C. Plasma was obtained within 2 hours from venipuncture by centrifugation at 2000 g for 20 min at 4°C, rapidly frozen and stored at −80°C. Serum total and HDL cholesterol, triglycerides, C-Reactive Protein (CRP), glycaemia and thyroid hormones were measured by standard biochemical assays. The concentration of serum LDL was calculated by using the Friedewald equation: LDL = total cholesterol – HDL – (triglycerides/5). Serum insulin was measured by a chemiluminescent immunoassay (LIAISON^®^ Insulin assay, DiaSorin, Saluggia, Italy) and analyzed by the LIAISON^®^ Analyzer. Insulin resistance status was assessed as homeostasis model assessment of insulin resistance (HOMA-IR) according to the following formula [67]: insulin (μU/mL) x glucose (mmol/L) / 22.5. Plasma levels of IL-6, IL-10, TNF-α, serum amyloid A (A-SAA), adiponectin, leptin, and resistin were measured by multiplex sandwich ELISA technology (SearchLight, Aushon Biosystems, Billerica, MA) according to the manufacturer's instructions. Samples, standards, and reagents were dispensed by a standardized technique employing a robotic liquid handling system with 16 channels (Microlab^®^ STAR, Hamilton Robotics, Reno, NV). Plasma TGF-β1 levels were determined by ELISA using a commercial kit (DRG Instruments GmbH, Marburg, Germany) according to the manufacturer's instructions and concentration of TGF-β1 was detected and quantified by a SynergyTM HT Multi-Detection Microplate Reader (Bio-Tek^®^ Instruments, Winooski, VT). Subjects who used medication that could influence the inflammatory status, such as steroid or nonsteroidal anti-inflammatory drugs and immunomodulant agents, during the week before blood drawn were excluded from adipokines and cytokines analysis.

### Statistical analysis

Demographic, lifestyle, anthropo-metric features and functional, cognitive, perceived health status as well as inflammatory status were examined in the two groups of subjects by univariate statistics (Independent samples *t*-test or Mann-Whitney U test, Chi-square test or Fisher's exact test) for continuous and categorical variables as appropriate. The dichotomous dependent variables were analyzed with logistic regression models and the odds ratio (OR) with 95% confidence interval was used as a measure of association. The regression model was adjusted for the effects of age, gender, education, marital status and BMI. All analyses were executed using SPSS 15.0 for windows (SPSS Inc., Chicago, IL, USA).

## SUPPLEMENTAL DATA TABLE


