# Supplementary material for: Centenarians’ offspring as a model of healthy aging: a reappraisal of the data on Italian subjects and a comprehensive overview
Source: Aging (Albany NY). 2016 Mar 12;8(3):510–9. doi: 10.18632/aging.100912 (PMC4833142; doi:10.18632/aging.100912)
Supplement: Supplementary file 1 [file aging-08-510-s001.pdf]

SUPPLEMENTAL DATA

Supplemental Table 1. Comparison of sample size, control selection and main results among this paper and the previous ones exploiting centenarians' offspring model

Study	Participants		Main results
Present study	267 Centenarians' offspring	107 Controls (age-matched offspring with both parents born in the same birth cohort of centenarians but dead before the threshold age over which subjects were classified as long-lived)	Centenarians' offspring had a lower prevalence of stroke, cerebral thrombosis-haemorrhage, hypertension, hypercholesterolemia, and other minor diseases, a lower BMI and waist circumference and a better functional and cognitive status (IADL and SMMSE scores, ability of walking 500 meter, going up and down the stairs, performing chair stand test and displaying higher grip strength).
Terry et al., 2003 [7]	177 Centenarians' offspring	166 Controls (offspring of parents born in the same years as the centenarians but at least one of whom died at average life expectancy)	Centenarians' offspring demonstrate a markedly reduced prevalence of cardiovascular disease and cardiovascular risk factors.
Barzilai et al., 2003 [18]	216 Ashkenazi Jewish Centenarians Offspring	258 Ashkenazi Jewish controls (75 spouses of the offspring and 183 age-matched Ashkenazi Jewish); 589 Caucasian controls (age-matched participants of the Framingham Study)	Centenarians' offspring had a larger HDL and LDL particles size. This phenotype is associated with a lower prevalence of hypertension, CVD, metabolic syndrome and increased homozygosity for the 14405V variant in CEPD.
Terry et al., 2004 [8]	177 Centenarians' offspring	166 Controls (offspring of parents born in the same years as the centenarians but at least one of whom died at average life expectancy)	Centenarians' offspring showed a marked delay in the age of onset for cardiovascular disease, diabetes, hypertension, and stroke but not for cancer, osteoporosis, and thyroid disease.
Terry et al,, 2004 [9]	295 Centenarians' offspring	276 Controls (offspring of parents born in the same years as the centenarians but at least one of whom died at average life expectancy)	Centenarians' offspring showed lower all-cause mortality rates and cause-specific mortality rates for cancer and coronary heart disease.
Atzmon et al., 2006 [19]	216 Ashkenazi Jewish Centenarians' Offspring	258 Ashkenazi Jewish controls (75 spouses of the offspring and 183 age-matched Ashkenazi Jewish)	Centenarians' offspring have an higher prevalence of homozygosity for the 641C allele in the APOC3 promoter. This genotype was associated with significantly lower serum levels of APOC3, a favorable pattern of lipoprotein levels and sizes and with a lower prevalence of hypertension and greater insulin sensitivity.
Adams et al., 2008 [16]	404 Centenarians' offspring	243 Controls (200 spouses of centenarian offspring and 43 individuals with at least one parent born at approximately the same time as the centenarians but who died at the average life expectancy for that birth cohort)	Centenarians' offspring had a lower risk of myocardial infarction, stroke, diabetes mellitus. Centenarian offspring were less likely to die during the follow-up.
